# Nitric Oxide and Biological Mediators in Pediatric Chronic Rhinosinusitis and Asthma

**DOI:** 10.3390/jcm8111783

**Published:** 2019-10-25

**Authors:** Valentina Agnese Ferraro, Stefania Zanconato, Eugenio Baraldi, Silvia Carraro

**Affiliations:** Women’s and Children’s Health Department, University of Padova, via Giustiniani 2, 35128 Padova, Italy

**Keywords:** nitric oxide, biological mediators, pediatric rhinosinusitis, pediatric asthma

## Abstract

Background: In the context of the so-called unified airway theory, chronic rhinosinusitis (CRS) and asthma may coexist. The inflammation underlying these conditions can be studied through the aid of biomarkers. Main body: We described the main biological mediators that have been studied in pediatric CRS and asthma, and, according to the available literature, we reported their potential role in the diagnosis and management of these conditions. As for CRS, we discussed the studies that investigated nasal nitric oxide (nNO), pendrin, and periostin. As for asthma, we discussed the role of fractional exhaled nitric oxide (feNO), the role of periostin, and that of biological mediators measured in exhaled breath condensate (EBC) and exhaled air (volatile organic compounds, VOCs). Conclusion: Among non-invasive biomarkers, nNO seems the most informative in CRS and feNO in asthma. Other biological mediators seem promising, but further studies are needed before they can be applied in clinical practice.

## 1. Introduction

The possible coexistence of rhinosinusitis and asthma is well known [1,2], in the context of the so-called unified airway theory, which describes the upper and lower airways as a single functional unit [3,4,5]. Moreover, the upper and lower respiratory tracts have common histological structures, including the basement membrane, lamina propria, ciliary epithelium, glands, and goblet cells [6].

Nowadays, more and more interest is directed to the study of noninvasive tests, which could help assess the presence and nature of airway inflammation in childhood chronic rhinosinusitis (CRS) and asthma, in order to learn more about the underlying pathological pathways of these complex diseases, and potentially guiding the development of a personalized medicine.

The better studied noninvasive marker of airway inflammation is nitric oxide (NO). NO is a free radical gas produced from L-arginine mainly by two enzymes: constitutive nitric oxide synthase (NOS), which constantly generates low concentrations of NO, and inducible NOS (iNOS), also called type 2 NOS, which is present in airway epithelial cells, where it is upregulated by proinflammatory cytokines, such as tumor necrosis factor and interleukin-1β, and by lipopolysaccharides of Gram-negative bacteria [7,8,9].

NO is released throughout the airways, and both the NO released from the upper respiratory tract (nasal NO, nNO) and the lower respiratory tract (fractional exhaled nitric oxide, feNO) can be measured.

Nasal NO can be measured with a non-invasive method based on the nasal aspiration, at a constant flow rate, from one naris with gas entrained via the other naris (transnasal flow in series) during velum closure, in order to prevent leak of nasal NO via the posterior velopharyngeal aperture and to reduce contamination of nasal gas with lower airway air [10,11]. Till now, no other recommended methods have been described, even if this method requires patient collaboration, and alternatives have been studied for children who cannot manage velum closure [12,13,14,15].

The standardized method to measure feNO is the single breath on-line (SBOL) method, which is non-invasive, rapid, repeatable, and reproducible. The subject has to inhale through the mouth to total lung capacity (TLC), then exhaling during velum closure (against a positive pressure of 5–20 cmH_2_O) [11,16]. This technique is well standardized in children who are able to cooperate, while until now, no standardized methods have been recommended in young or uncooperative children [17,18,19]. In these children, the most appropriate method is the tidal breathing offline method, which is carried out collecting exhaled air in an appropriate reservoir for later analysis [20,21,22]. One limit of this method is the lack of control in the expiratory flow, being feNO values highly flow-dependent. To overcome this problem, fast-response chemiluminescence analyzers and flow control devices have been developed [23], as well as mathematical algorithms, which try to obtain, from tidal breathing feNO values, the corresponding single breath flow values [24]. The second limit of the tidal breathing offline method is the contamination from nose-derived air, which contains higher NO levels that may affect the measurements of feNO; for this reason, the use of facemasks with a septum that separates the air from upper and lower airways has been suggested [22].

**Figure 1:**
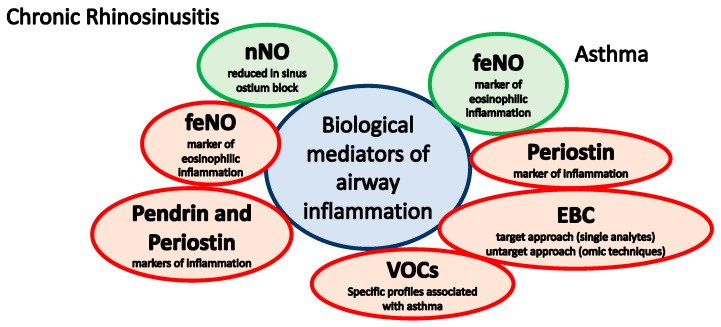
Biological mediators in pediatric rhinosinusitis and asthma. Green: applicable to clinical practice; Red: not applicable to clinical practice yet. nNo: nasal nitric oxide; feNO: fractional exhaled nitric oxide; EBC: exhaled breath condensate; VOCs: volatile organic compounds.

Here, we discussed the possible role of NO as a biomarker in chronic rhinosinusitis (CRS) and asthma in children. Moreover, we described the main other biological mediators that have been evaluated in these conditions. In particular, the possible role of periostin and pendrin was reported, and the potential of molecules measured in exhaled air (volatile organic compounds, VOCs) and exhaled breath condensate was discussed. Figure 1 summarizes these biomarkers, highlighting the current applicability in clinical practice.

## 2. Chronic Rhinosinusitis

Even if the diagnosis of CRS in children is based on symptoms (nasal obstruction, nasal discharge, facial pain/pressure, and reduced/lost smell) persisting for more than 12 weeks with associated endoscopic or radiographic findings [25], a number of biomarkers has been studied for their possible role in diagnosis, work-up, and management of this condition [2].

### 2.1. Nitric Oxide

NO has been extensively studied in CRS, mainly because upper airways represent the main source of respiratory NO [26,27], and, additionally, paranasal sinuses are the main production site [28]. In the upper airways, NO has several functions: a specific host defense against infective agents (bacteria, viruses, and fungi) [29], modulator of cilia motility [30], a regulator of nasal airflow humidification and warming [31], airborne messenger between higher and lower respiratory tracts [32,33].

The role of nasal NO in sinusitis has been evaluated in some studies. The first study describing variations of nasal NO levels during acute sinusitis was published in 1997 by Baraldi et al. [34]. The authors showed that children with acute maxillary sinusitis had a marked reduction in nasal NO concentrations, and nNO returned to normal levels after antibiotic therapy. This finding could be caused by mechanical obstruction of the draining ostia and by negative pressure within the sinuses, resulting in a decreased passage of NO from sinuses to the nasal lumen [34]. In keeping with this, in adult patients, it was demonstrated that despite patients with CRS with nasal polyps had high level of NOS2 in nasal epithelium, because of the inflammation of nasal and paranasal cavities, they exhibited lower nNO compared to patients with uncomplicated allergic rhinitis; moreover, the higher was the extent of polyposis and the lower were the levels of nNO [35]. This was probably due to the osteomeatal complex obstruction, which was associated with the inability of NO produced in the sinuses to reach the nasal cavity [35]. Likewise, another study confirmed, in patients with polyposis (which lead to blockade of the osteomeatal complex), a negative correlation between nNO and the degree of sinus disease [36]. In keeping with this, in a patient with CRS and polyposis, nNO diminished with the increase of sinus opacification observed with CT scans [37]. Even if these studies were mostly performed in adults (Table 1 summarizes these studies), they pointed out that inflammation of sino-nasal mucosa, especially if associated with polyps, prevents NO to flow from sinuses to the nasal lumen, so that reduced nNO levels are measured in these conditions.

Noteworthy, the reduction of nasal NO may lead to a vicious circle, increasing the risk of recurrent infections in these subjects, since NO plays a role in host airway defenses against exogenous agents [38].

In conclusion, although the clinical relevance of finding reduced nNO levels in CRS is limited [8,39], this biomarker can be useful to monitor sinus ostium block during both post-medical and postsurgery follow-up in subjects affected by bilateral nasal polyposis [8].

Even if feNO is considered a marker of lower airway eosinophilic inflammation, in the context of the previously described unified airway theory, recent studies have analyzed this marker in CRS, with variable results. Kobayashi et al. showed that patients with eosinophilic CRS without asthma did not have high feNO levels, while feNO levels were elevated in well-controlled asthmatic patients with eosinophilic CRS [40]. On the other hand, Zhang et al. showed that 29% of the patients with CRS with nasal polys, without pulmonary disease, had increased feNO [41], and Jeong et al. showed that 30 non-asthmatic, non-atopic patients with CRS with nasal polyps had a significantly higher feNO than healthy controls [42]. Similarly, Takeno et al. analyzed 33 patients with CRS with nasal polyps and found high feNO (defined as feNO >25 ppb) in 22 (66%), 8 (36%) of which with no history of asthma [43]. Furthermore, recent studies demonstrated that in patients with eosinophilic CRS, feNO levels correlated with the severity of the CT findings [40,44], and a reduction of this biomarker had been described after functional endoscopic sinus surgery [44].

In conclusion, the available data on feNO levels in CRS is not unanimous, and for the time being, there are no clear recommendations for its clinical use.

### 2.2. Pendrin and Periostin

Pendrin is an ion exchanger involved in inflammation and mucus production in patients with CRS, as well as in asthmatic patients [45,46]. Its role in mucus production is not only due to a direct effect but also mediated by the recruitment of inflammatory cells [46]. Pendrin has also been shown to regulate epithelial air-surface liquid levels and composition [45,47].

It has been demonstrated that pendrin is overexpressed in the sinonasal tissue, including epithelial cells and submucosal gland cells, in patients with CRS and nasal polyps, suggesting a pathogenetic role for this molecule [48]. The increased levels of pendrin might contribute to chronic inflammatory response, mucus production, and decreased mucociliary clearance [49].

Periostin is an extracellular matrix protein, a highly inducible product of IL-4 or IL-13, which are signature cytokines of the Th2-type immune response [50,51]. Moreover, periostin plays an important role as a regulator of fibrosis and collagen deposition [52]. Recently, the analysis of sinonasal mucosal biopsies, obtained from CRS patients, showed that periostin was associated with the presence of basement membrane thickening, fibrosis, and tissue eosinophilia and might identify patients undergoing remodeling changes [53]. In addition, its overproduction in the nasal mucosa of patients with CRS has been suggested to contribute to polyp formation [48,54,55]. Xu et al. demonstrated that periostin and VEGF (vascular endothelial growth factor) were higher in eosinophilic nasal polyps than in non-eosinophilic nasal polyps and control tissue, and in vitro VEGF was upregulated by periostin, suggesting that periostin might play an important role in the development of eosinophilic nasal polyps [56]. It has been shown that serum periostin in combination with blood eosinophils and basophils count has the potential to discriminate eosinophilic nasal polyps and non-eosinophilic nasal polyps [57] and, in combination with IgE and *Staphylococcal enterotoxin* (SE)-IgE, may be useful to identify nasal polyps with moderate and severe type 2 inflammation [58].

In conclusion, the analysis of pendrin and periostin can provide some insights into the pathogenetic mechanisms involved in CRS. Nonetheless, such measurements are still limited to research, and they do not have a role in clinical practice yet.

## 3. Asthma

In the past two decades, many studies in the field of asthma focused on the investigation of biomarkers relevant for the diagnosis, phenotyping, or treatment of the disease [59,60,61]. Here, we discussed the most widely studied biomarkers, focusing on those measured non-invasively, since this aspect is particularly important when dealing with children

### 3.1. Fractional Concentration of Exhaled Nitric Oxide

Four years after the first report on the presence of nitric oxide in exhaled human breath [62], an increase in its levels has been reported in children with asthma [63], in particular during asthma exacerbation with a rapid decline after oral steroid therapy [64]. Subsequently, several studies analyzed feNO in pediatric asthma, demonstrating its role as a marker of eosinophilic airway inflammation, since it is correlated with eosinophil counts in blood and induced sputum or bronchoalveolar lavage fluid, with serum eosinophil cationic protein and with IgE levels [9,65,66,67]. Recently, it has been described a fair diagnostic accuracy of feNO for identifying asthmatic patients [68] and, as recently suggested by Pavord et al., for identifying the treatable trait of eosinophilic asthma (e.g., to identify patients who are likely to benefit from inhaled corticosteroids) [61]. On the other hand, being feNO increased also in other atopic conditions, other authors suggested that low feNO levels predict a non-eosinophilic asthma phenotype better than high levels can predict an eosinophilic one [69,70].

As far as it concerns young wheezing children, several studies demonstrated that feNO levels were higher in those with recurrent wheezing compared to healthy controls [71,72,73], in those with frequent wheezing with high asthma predictive index (API) compared to low API [74,75], and in those with persistent wheezing compared to transient wheezing [76]. Therefore, it has been suggested that feNO could help phenotype preschool-age children with recurrent wheezing, contributing to the identification of those with early-onset asthma. In detail, it has been demonstrated that in high-risk preschool children (at a mean age of 22 months), high feNO levels were associated with increased risk for school-age asthma [77], and in preschool children with symptoms suggestive of asthma, both feNO and specific IgE to inhalant allergens were associated with asthma at 8 years [78]. In addition, in longitudinal cohort studies in infants and toddlers (<2 years) with recurrent wheezing, feNO values higher than or equal to 30 ppb had a high predictive value for persistent wheezing at 3 years of age [79], and an increase in feNO was associated with a decrease in lung function 6 months later [80].

The predictive value of feNO for the development of asthma was analyzed even in healthy school children, showing that children in the highest feNO quartile had an increased risk of developing asthma compared to those with the lowest quartile [81]. Also, a study carried out in children (mean age 8.4 years, follow-up 5 years), with allergic rhinitis (without asthma) and feNO > 35 ppb at baseline, demonstrated a higher risk of new-onset asthma and a higher decrease in lung function, suggesting less lung growth in children with high feNO values [82].

feNO is also a marker of inhaled corticosteroids responsiveness, as well as a possible marker of treatment compliance since several studies demonstrated a drop in its levels in response to steroid therapy [71,83,84]. A recent systematic review and meta-analysis showed that adjusting treatment, according to feNO levels, reduced the likelihood of asthma exacerbations at the expense of increased inhaled corticosteroids doses [85].

In conclusion, feNO has been studied in particular for its potential role in eosinophilic asthma detection, early asthma identification, and corticosteroid responsiveness prediction. These possible clinical applications are well summarized in the available international guidelines (Table 2):

- The National Institute for Health and Care Excellence (NICE, 2017) recommends measuring feNO (described as positive test when more than or equal to 35 ppb) in children (aged 5 to 16 years) with symptoms suggestive of asthma, if there is diagnostic uncertainty after initial assessment (e.g., normal spirometry or obstructive spirometry with a negative bronchodilator reversibility test) [86]. Furthermore, using feNO to monitor asthma control is not routinely recommended [86].
- The 2019 British Thoracic Society guidelines recommend to use feNO (if available) to find evidence of eosinophilic inflammation (regard a feNO level of 35 ppb or more as a positive test), keeping in mind that a positive test increases the probability of asthma, but a negative test does not exclude asthma [87]. Also, except in specialist asthma clinics, the routine use of feNO testing to monitor asthma in children is not recommended [87].
- The 2019 Global Initiative of Asthma (GINA) guidelines report that feNO is not useful for ruling in or ruling out a diagnosis of asthma nor for guiding asthma treatment in the general population. Among alternative strategies for adjusting asthma treatment in children, GINA guidelines report that feNO-guided treatment significantly reduces exacerbation rates compared with guidelines-based treatment (Evidence A). Furthermore, feNO seems to be a useful adjunct in diagnosing asthma in pre-school children with recurrent wheezing, in whom an elevated feNO (recorded 4 weeks from any URTI) predicts asthma at school age [88].

### 3.2. Periostin

As previously described, periostin is an extracellular matrix protein upregulated by classic type 2 cytokines IL-4 and IL-13 [50,51], which was described in several reports as a useful biomarker of T2-inflammation in adult asthmatic patients [89,90,91]. Moreover, in asthmatic patients, periostin plays an important role as a regulator of fibrosis, airway remodeling, collagen deposition, and mucus production from goblet cells [50,52,92,93].

Despite an increased periostin level described in children with asthma [94,95], periostin is unlikely to be a useful biomarker of type 2 inflammation in children, mainly because its levels increase due to bone growth and this may overlap with local production within the airways [93,96].

In conclusion, as in CRS, also in asthma, the study of periostin is currently limited to the field of research.

### 3.3. Exhaled Breath Condensate (EBC)

Among non-invasive methods for studying airway inflammation, exhaled breath condensate (EBC) is one of the most attractive. It is a biofluid collected during tidal breathing by cooling exhaled air by contact with a cold surface or condenser [83,97]. The condensate contains unstable volatile and semi- and non-volatile molecules, and its composition is thought to mirror that of the airway lining fluid; EBC is considered a promising biofluid, which allows the noninvasive study of pulmonary biochemical and inflammatory processes [83,98,99,100]. Many studies have investigated the possible role of EBC analysis in asthma, using both targeted (a measurement of single analytes) and untargeted (omic techniques) approaches.

Through a targeted approach, many single mediators related to inflammation and oxidative stress have been searched in EBC. Among them, the most relevant are:

- pH, which tended to be lower in children with severe or acute asthma but not in mild and stable disease [101,102,103];
- Leukotrienes (LT): LTB4, a potent inflammatory mediator and a chemoattractant for neutrophils, was increased in the EBC of asthmatic children, being twice as high in steroid-naïve patients with asthma as in healthy subjects [104,105]; Cysteinyl leukotrienes (LTC4, LTD4, and LTE4), powerful constrictors and proinflammatory mediators, were increased in particular in unstable or severe asthma [106,107,108];
- 8-isoprostane, hydrogen peroxide (H_2_O_2_), and other markers of oxidative stress, which were increased in asthma [107,109]; in particular, H_2_O_2_ correlated with disease severity, disease control, and response to steroid treatment [110,111];
- 3-nitrotyrosine (3-NT) and other nitric oxide metabolites that were more concentrated in the EBC of asthmatic children than in healthy controls [102,112,113].

Since no single biomarker can fully describe the pathogenic processes underlying complex chronic diseases, “-omic approaches” have been applied to study the overall biochemical-metabolic composition of exhaled breath condensate, with the potential for identifying analyte profiles characteristic of specific conditions [98,99,114,115]. Both proteomics and metabolomics have been applied to EBC in asthma research.

Proteomics is defined as the study of the complete assessment of proteins in a biological sample in order to identify potential biomarkers associated with a specific disease [114]; therefore, detecting distinct protein biomarkers in different pathologies may assist in disease diagnosis, monitoring, treatment, and prognosis [116]. The complexity of proteomics, due to alternative splicing, posttranscriptional, and translational modifications and the enormous dynamic range of protein concentrations in biological samples, makes this research field one of the most interesting in the last years, even if still an object of speculation [117]. As far as it concerns healthy subjects, several studies explored EBC in order to characterize their protein composition (proteome maps), which could be useful for future clinical studies dedicated to the discovery of novel protein biomarkers for pulmonary diseases [118,119]. In keeping with this, Bloemen et al. found a specific pattern of expressed peptides in asthmatic children [120].

Metabolomics, without any priori hypothesis, studies the metabolite composition (or metabolome) of a biological sample, using a spectroscopic technique (usually NMR spectroscopy and mass spectrometry). It is nowadays considered the “-omic” science that comes closer to phenotype expression because the metabolome is the result of both genetic influences and environmental stimuli [121,122]. Therefore, metabolomics provides a snapshot of the overall physiology of the host and its response to the environment [121]. In the EBC of asthmatic children, the metabolomic analysis was applied to characterize the airway biochemical fingerprints, enabling the discrimination of children with and without asthma [123]. Furthermore, in children with asthma, EBC metabolomic analysis distinguished different asthma phenotypes and enabled the identification of a specific profile associated with severe asthma [124].

In conclusion, the analysis of EBC, both using targeted and untargeted approaches, seems promising for the study of physio-pathological mechanisms underlying asthma. Nonetheless, despite two comprehensive Task Force reports of the European Respiratory Society (ERS) and American Thoracic Society (ATS) published in 2005 [100] and in 2017 [83], EBC-analysis is not yet routinely applicable in clinical setting, mainly, because of the poor reproducibility of biomarkers and the absence of large surveys for determination of reference-normal values [125]. In keeping with this, a recent review published by Bannier et al. showed that studies on EBC research in pediatric asthma, performed between 2013 and 2018, are hardly comparable due to large heterogeneity in study populations, study methods, EBC collection methodologies, EBC biomarkers, analytical methods, and limits of detection [126].

### 3.4. Volatile Organic Compounds (VOCs)

In the last decades, exhaled breath has been studied using a metabolomic approach in the so-called “breathome”, that is the fingerprint of volatile organic compounds (VOCs) [127]. Airway VOCs are organic chemicals (e.g., a chemical compound that contains carbon) originated from the upper and lower airways and also from the capillary bed near the alveoli [128]. In order to collect VOCs from exhaled breath, different methodological approaches have been studied, taking care to exclude organic compounds from ambient air, to apply the correct breathing maneuvers, and to use the most suitable sampling materials [128].

Two different techniques have been used to study exhaled VOC profiles: (a) gas chromatography with mass spectrometry, a quantitative method that identifies individual components, and (b) the electronic Nose (e-Nose), a qualitative method that obtains probabilistic discrimination between biomarker profiles [129,130].

In pediatric asthma, different VOCs profiles have been described in children with and without asthma [131,132,133,134,135]. The analysis of exhaled VOCs may contribute to asthma diagnosis [136] and the discrimination of asthmatic children from those with transient wheezing symptoms [137]. Also, several studies demonstrated the potential role of VOCs analysis in the prediction of asthma exacerbation [138,139] and in the characterization of children with persistently controlled and uncontrolled asthma [140]. On the contrary, recently, Bannier et al. reported that Aeonose (an easy-to-use hand-held eNOse) used in children ≥6 years had high feasibility (>98% successful measurements), but a modest diagnostic accuracy for the discrimination between asthma and healthy controls [141].

In conclusion, VOCs analysis is an attractive non-invasive method that could contribute to the identification of asthmatic subjects, even if larger studies are needed, in order to standardize the procedures and validate the technique of sampling and analysis.

## 4. Conclusions

Several non-invasive biomarkers have been investigated to study inflammation in CRS and asthma. As far as it concerns CRS, there is clear evidence that in subjects with bilateral nasal polyposis, nNO is reduced because of sinus ostium block; other biomarkers have been studied in this condition (in particular, pendrin and periostin), but, nowadays, they have no clear role in clinical practice.

In pediatric asthma, feNO levels may have a role in the characterization of Th2-mediated eosinophilic inflammation in the early identification of asthma in pre-school children with recurrent wheezing and the prediction of steroid responsiveness. Nonetheless, the use of feNO measurements in clinical practice is still limited, as specified by the current international guidelines.

Eventually, even if several studies investigated the possible role of EBC and VOCs analysis in pediatric asthma, they are not ready for clinical practice yet, and larger studies are needed to standardize the procedures of sampling and analysis.

## Figures and Tables

**Table 1:** List of studies that evaluated nasal nitric oxide (nNO) in rhinosinusitis.

Study	Aims	Population	Results (mean ± SD)	Conclusions
[34]	To evaluate nNO in children with acute maxillary sinusitis before and after treatment with antibiotic therapy	16 children (4–13 years) with acute maxillary sinusitis; 16 age- and sex -matched healthy control subjects	(1) nNO = 70 ± 8.7 ppb sinusitis before antibiotic therapy; (2) nNO = 220 ± 15 ppb sinusitis after antibiotic therapy (amoxicillin/clavulanate); (3) nNO = 245 ± 15 ppb healthy control subjects	During acute maxillary sinusitis, nNO is decreased; nNO returns to normal after antibiotic therapy
[37]	To examine if nNO is affected by paranasal sinus inflammatory diseases	20 patients with nonallergic nasal polyposis (age 48 ± 3 years); 42 control subjects (age 42 ± 3 years)	(1) nNO = 150 ± 20 ppb in patients with nasal nonallergic polyposis; (2) nNO = 223 ± 6 ppb in controls	nNO in patients with nasal polyposis is decreased compared to controls, and it depends on the degree of obstruction of the paranasal sinuses
[35]	To evaluate nNO in patients with nasal polyposis compared with allergic rhinitis and to analyze the effect of polyp treatment on nNO	44 patients with rhinitis without polyps (age = 39 ± 13.6 years) and 38 with polyps (age = 45.6 ± 4.5 years); 20 normal controls (age = 36.9 ± 11.6 years); 23 patients with polyposis pre- and post-treatment (age = 48.8 ± 4.2 years)	(1) nNO = 740.9 ± 148.1 ppb in normal controls (2) nNO = 659.8 ± 304.8 ppb in allergic rhinitis (3) nNO is significantly lower in patients with polyps than allergic rhinitis without polyps (Kruskal–Wallis, *p* = 0.0001, x^2^ = 37.6, d.f. = 4) (4) Successful treatment, with reduction in polyp volume, associated with a rise in NO levels (*p* = 0.042)	nNO levels are low in nasal polyps. A rise in nNO is seen with successful polyp treatment
[36]	To study the effect of CRS therapy on nNO and to see whether nNO changes correlate with other assessments.	90 patients (mean age 43 ± 13 years) with CRS who still had troublesome symptoms after initial therapy with dexarhinaspray and nasal douching	(1) Baseline nNO correlate with CT scores (*p* < 0.001) (2) The mean nNO levels for the three grades of severity at CT scan are grade1 (less severe) 537 ± 202, grade 2 362 ± 188, and grade 3 165 ± 151 ppb. (3) The percentage rise in nNO correlates with changes in symptom scores (*p* < 0.001), saccharin clearance time (*p* < 0.001), endoscopic changes (*p* < 0.001), polyp grades (*p* < 0.05 at 6 months, *p* < 0.01 at 12 months), and surgical scores (*p* < 0.01).	nNO provides a valuable non-invasive objective measure of the response of CRS to therapy

ppb: parts per billion; CRS: chronic rhinosinusitis; nNO: nasal nitric oxide.

**Table 2:** How guidelines consider the use of fractional exhaled nitric oxide (feNO) as a biomarker of asthma ().

Guideline	Cut-off Value	How to Use feNO in Clinical Practice
[86]	feNO positive if more than or equal to 35 ppb	in children (aged 5 to 16 years) with symptoms suggestive of asthma, if there is diagnostic uncertainty after the initial assessment not routinely recommended to monitor asthma control
[87]	feNO positive if more than or equal to 35 ppb	(if available) to find evidence of eosinophilic inflammation a positive test increases the probability of asthma, but a negative test does not exclude asthma the routine use of feNO testing to monitor asthma in children is not recommended, except in specialist asthma clinics
[88]	No clear cut-off value	feNO is not useful for ruling in or ruling out a diagnosis of asthma feNO is not useful for guiding asthma treatment in the general population, even if among alternative strategies for adjusting asthma treatment in children; feNO-guided treatment significantly reduces exacerbation rates compared with guidelines-based treatment (Evidence A)

feNO = fractional exhaled nitric oxide; ppb = parts per billion.
